# Role of *BraRGL1* in regulation of *Brassica rapa* bolting and flowering

**DOI:** 10.1093/hr/uhad119

**Published:** 2023-06-02

**Authors:** Yudan Wang, Shiwei Song, Yanwei Hao, Changming Chen, Xi Ou, Bin He, Jiewen Zhang, Zhehao Jiang, Chengming Li, Shuaiwei Zhang, Wei Su, Riyuan Chen

**Affiliations:** Key Laboratory of Horticultural Crop Biology and Germplasm Innovation in South China, Ministry of Agriculture, College of Horticulture, South China Agricultural University, Guangzhou 510642, China; Key Laboratory of Horticultural Crop Biology and Germplasm Innovation in South China, Ministry of Agriculture, College of Horticulture, South China Agricultural University, Guangzhou 510642, China; Key Laboratory of Horticultural Crop Biology and Germplasm Innovation in South China, Ministry of Agriculture, College of Horticulture, South China Agricultural University, Guangzhou 510642, China; Key Laboratory of Horticultural Crop Biology and Germplasm Innovation in South China, Ministry of Agriculture, College of Horticulture, South China Agricultural University, Guangzhou 510642, China; Key Laboratory of Horticultural Crop Biology and Germplasm Innovation in South China, Ministry of Agriculture, College of Horticulture, South China Agricultural University, Guangzhou 510642, China; Key Laboratory of Horticultural Crop Biology and Germplasm Innovation in South China, Ministry of Agriculture, College of Horticulture, South China Agricultural University, Guangzhou 510642, China; Key Laboratory of Horticultural Crop Biology and Germplasm Innovation in South China, Ministry of Agriculture, College of Horticulture, South China Agricultural University, Guangzhou 510642, China; Key Laboratory of Horticultural Crop Biology and Germplasm Innovation in South China, Ministry of Agriculture, College of Horticulture, South China Agricultural University, Guangzhou 510642, China; Key Laboratory of Horticultural Crop Biology and Germplasm Innovation in South China, Ministry of Agriculture, College of Horticulture, South China Agricultural University, Guangzhou 510642, China; Key Laboratory of Horticultural Crop Biology and Germplasm Innovation in South China, Ministry of Agriculture, College of Horticulture, South China Agricultural University, Guangzhou 510642, China; Key Laboratory of Horticultural Crop Biology and Germplasm Innovation in South China, Ministry of Agriculture, College of Horticulture, South China Agricultural University, Guangzhou 510642, China; Key Laboratory of Horticultural Crop Biology and Germplasm Innovation in South China, Ministry of Agriculture, College of Horticulture, South China Agricultural University, Guangzhou 510642, China

## Abstract

Gibberellin (GA) plays a major role in controlling *Brassica rapa* stalk development. As an essential negative regulator of GA signal transduction, DELLA proteins may exert significant effects on stalk development. However, the regulatory mechanisms underlying this regulation remain unclear. In this study, we report highly efficient and inheritable mutagenesis using the CRISPR/Cas9 gene editing system in *BraPDS* (phytoene desaturase) and *BraRGL1* (key DELLA protein) genes. We observed a loss-of-function mutation in *BraRGL1* due to two amino acids in GRAS domain. The flower bud differentiation and bolting time of *BraRGL1* mutants were significantly advanced. The expression of GA-regulatory protein (*BraGASA6*), flowering related genes (*BraSOC1*, *BraLFY*), expansion protein (*BraEXPA11*) and xyloglucan endotransferase (*BraXTH3*) genes was also significantly upregulated in these mutants. *BraRGL1*-overexpressing plants displayed the contrasting phenotypes. *BraRGL1* mutants were more sensitive to GA signaling. BraRGL1 interacted with BraSOC1, and the interaction intensity decreased after GA_3_ treatment. In addition, BraRGL1 inhibited the transcription-activation ability of *BraSOC1* for *BraXTH3* and *BraLFY* genes, but the presence of GA_3_ enhanced the activation ability of *BraSOC1*, suggesting that the BraRGL1-BraSOC1 module regulates bolting and flowering of *B. rapa* through GA signal transduction. Thus, we hypothesized that BraRGL1 is degraded, and BraSOC1 is released in the presence of GA_3_, which promotes the expression of *BraXTH3* and *BraLFY*, thereby inducing stalk development in *B. rapa*. Further, the *BraRGL1-M* mutant promoted the flower bud differentiation without affecting the stalk quality. Thus, *BraRGL1* can serve as a valuable target for the molecular breeding of early maturing varieties.

## Introduction

Gene editing techniques are useful in studies investigating gene function and crop enhancement strategies. CRISPR/Cas9 is an emerging, rapidly evolving, and powerful gene-editing technology. Compared with established gene editing technologies, such as zinc finger nucleases and transcription activator-like effector nucleases [1, 2], CRISPR/Cas9 technology is increasingly favored by researchers due to its advantages including simplicity of use, cost-effectiveness, fast operation, targeted mutation, simultaneous editing of multiple target genes, homozygous mutants in the T_0_ generation, high specificity, and easy mutation detection [3]. To date, the CRISPR/Cas9 system has been established in numerous plants, including *Arabidopsis* [4], tobacco [5], sorghum [6], wheat [7], rice [3], *Zea mays* [8], tomato [9], cucumber [10], banana [11], chrysanthemum [12], kiwifruit [13], *Brassica carinata* [14], switch grass [15]. To address the low efficiency of CRISPR/Cas9 at multi-gene or multi-site editing, researchers combined tRNA and gRNA to form a polycistronic gene, and established tandem two or more sgRNAs on the same expression vector, thus generating a large number of sgRNAs carrying the correct targeting sequence in order to greatly improve mutation efficiency [16]. This system has been utilized successfully in rice, corn, wheat, and other crops [16–18], which has greatly promoted the genetic research of plants and improved crop varieties.

*Brassica* vegetables are important agricultural and horticultural crops. However, only a few instances of effective genome editing in *Brassica* vegetables have been documented [14, 19–22]. One of these cases was *BcPME37c* gene knockout in ‘Youqing 49’ [14, 19–22]. A genome-wide triplication event that occurred in *Brassica rapa* during evolution produced multicopy genes or numerous substantially related homologous genes [23]. However, research on gene function and molecular breeding in *B. rapa* is severely constrained due to the difficulty of its genetic transformation compared to that in other *Brassica* species. *Brassica oleracea* and *B. rapa* have a close genetic association. The efficient editing of the cabbage genome by Ma *et al.* serves as a model for the development of an effective gene editing technology system in *B. rapa* [24].

Stalks are the main food product of Caixin*.* Bolting (stem thickening and elongation) and flowering are important stalk developmental traits, both of which are directly related to plant yield and quality [25]. Exogenous gibberellin (GA_3_) treatment advances the timing of bolting and flowering in Caixin because GA is the primary regulator of these processes [26, 27] by acting through the GA signaling pathway. As a negative regulator of GA signal transduction, DELLA protein is a key factor in modulating the GA response [28]. DELLA proteins are distinguished by a GRAS domain and a DELLA/TVHYNP motif at the N-terminus [29]. DELLA proteins work as nuclear-localized transcriptional regulators, and their accumulation is heavily reliant on the concentrations of GA present within the cell. Increased GA concentrations encourage the polyubiquitination of DELLAs by the 26S proteasome and the GIBBERELLIN INSENSITIVE DWARF1 (GID1) receptor [29–31].

One of the most important functions of DELLA is to regulate plant height. Many DELLA mutants have been identified and most of them are insensitive to GA signals and exhibit dwarfing and delayed flowering, including *gai* in *Arabidopsis* [32], *Rht* in wheat [33], *sln1d* in barley [34]. Another phenotype of DELLA mutants is a GA-sensitive slender form, whose product does not appear to be able to repress, often referred to as a loss-of-function mutation, including *rga* and *rgl* in *Arabidopsis* [35, 36], *slr* in rice [37], and *sln1c* in barley [34]. The molecular mechanism of the DELLA protein in *B. rapa* may be studied thanks to advancements in studies on its function and GA signal transduction pathways in model plants [38]. We previously isolated five DELLA family genes (RGA1, RGL1, RGA2, RGL2, and RGL3) from Caixin and examined their expression levels in two distinct cultivars. Only one of these, *BraRGL1* (*BraA02g017510.3.5C*), showed significantly varied expression levels at the two-true-leaf stage, suggesting that it may play a role in how various types of early bud differentiation processes affect bolting and flowering [39]. However, its specific functions have not yet been verified.

Vegetative and reproductive growth are synchronous during stalk formation, which is different from the development of *Arabidopsis*, thereby making the stalk development in Caixin special and complex. Therefore, the establishment of an efficient genome-editing system for Caixin is of great significance for exploring the regulatory mechanisms involved in stalk development. Here, we achieved efficient editing of two genes (*BraPDS* and *BraRGL1*) and characterized the function of *BraRGL1*. We observed that the loss of function of *BraRGL* promoted early bud differentiation and bolting and was more sensitive to GA_3_. Protein–protein interaction analyses showed that BraRGL1 interacted with BraSOC1, and exogenous GA_3_ treatment weakened this interaction. In addition, we determined that the RGL1-SOC1 module regulated bolting and flowering in Caixin by controlling the expression of cell elongation and flower-related genes. These findings provide theoretical and technical support for us to further exploration of the regulatory mechanisms of stem development in *B. rapa*.

## Results

### Analysis of the *BraPDS* mutation efficiency and types

*PDS* encodes a key enzyme involved in carotenoid production. The albino phenotype caused by its disruption is straightforward to identify [5]. Therefore, we first selected *BraPDS* as a target to examine the effectiveness of genome modification using the tRNA-processing system in Caixin. Approximately 1800 explants were transformed using an *Agrobacterium*-mediated method with a vector containing the sgRNA-*BraPDS*-1234 cassette, and 22 T_0_ lines were generated. Among them, 16 lines exhibited the completely albino or mosaic albino phenotype, resulting in 72.72% knockout efficiency (Figure S1, Table S1; see online supplementary material). Full-length sequences of *BraPDS* gene in three albino buds and one albino plant (M1, M2, M3, and M4) were amplified by PCR, and the results were immediately sequenced. Four of the transgenic lines were heterozygous or chimeric mutants with overlapping peaks at the target site of *BraPDS* gene. We investigated the mutation types and frequencies by performing TA cloning and Sanger sequencing of the PCR products of the four lines. A total of 32 clones were randomly selected for sequencing, among which only four clones had the same wild type (WT) sequence, and all the other clones (87.5%) presented mutations at the target site (Figure S2a, see online supplementary material). All mutations were short insertions or deletions, with the 1 bp insertion being the most prevalent mutation type (Figure S2a and S3, see online supplementary material). The highest mutation frequency of the four target sites was site 4, with up to 71.88%, followed by site 2 with 50.0%, site 3 with a mutation frequency of 6.25%, and no mutagenesis was detected at site 1 (Table S1, see online supplementary material).

The mutated nucleotide sequences were translated into amino acid sequences to investigate mutations at the translational level (Figure S2b, see online supplementary material). All nine mutation types resulted in frameshift mutations that finally caused premature translation termination and gave rise to proteins with only 134, 257, and 260 amino acids in length (Figure S2b, see online supplementary material). The Phytoene desaturase domains of *BraPDS* were destroyed by each of the aforementioned mutations (Figure S2b, see online supplementary material). The loss of *BraPDS* gene function in M4 plants caused not only the albino phenotype, but also pollen development defects (Figure S1e, see online supplementary material). No off-target mutation was detected at the top two ranking off-targets (Figure S4, see online supplementary material). These findings suggest that CRISPR/cas9-induced target mutations are highly efficient and specific in *B. rapa.*

### Analysis of the *BraRGL1* mutation efficiency and types

**Figure 1:**
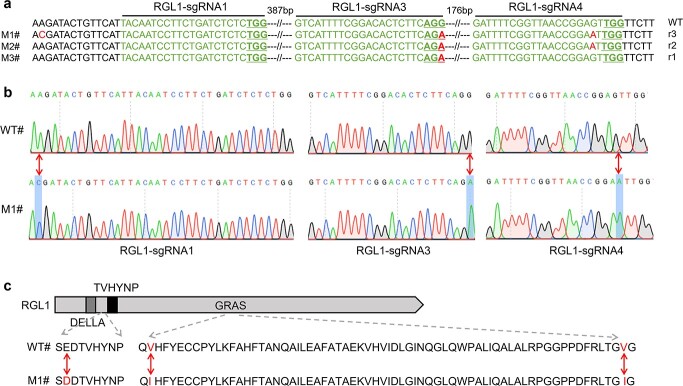
Mutagenesis types of *BraRGL1-*mutated lines. **a** Nucleotide sequence alignment of target sites in wild type (WT) and three *BraRGL1* mutants (M1, M2, and M3). The PAM sequence is underlined. Green indicates the target sequences; red indicates mutated bases. **b** Chromatogram of WT and three homozygous mutants at target sites and flanking sequences in *BraRGL1*. **c** Protein sequence alignment between WT and M1 mutants. Red arrows represent substitutions.

Our earlier research suggested that *BraRGL1* might be crucial for bolting and flowering in Caixin in various cultivar variations [39]. We further investigated the biological function of *BraRGL1* by knocking out the *BraRGL1*. Approximately 2000 explants were transformed to generate 19 T_0_-positive lines. Full-length of *BraRGL1* gene in all positive strains was amplified by PCR, and the products were directly sequenced. Twelve transgenic lines were homozygous or heterozygous mutants with base substitutions at target sites and flanking sequences in *BraRGL1*, resulting in 63.15% knockout efficiency (Table S1, see online supplementary material). Three strains (M1, M2, and M3) were randomly selected for cloning and sequencing. Half of these clones were consistent with the WT, whereas the other half presented mutations at the target sites and their flanking sequences. All mutation types were base substitutions and mainly occurred at target sites 3 and 4. No mutagenesis was detected at site 2 (Figure 1a and b; Table S1, see online supplementary material), which differed from the *BraPDS* gene modification.

Translation of the mutated DNA sequences into amino acid sequences and all three nucleotide substitutions resulted in amino acid mutations (Figure 1c). Notably, there was a mutation site between the DELLA and the TVHYNP domains where glutamate (E) was mutated to aspartic acid (D). And there were two amino acid mutations in the GRAS region of the C-terminus, two valines (V) mutated to isoleucine (I). The number of amino acids between the DELLA and TVHYNP domains is essential for GA signal reception. The GRAS domain is a functional structural region and controls DELLA protein activity [40, 41]. Considering the importance of these three mutation sites, we selected the M1 mutants for further analysis. The genotypes of the M1 offspring (T_0_ line) were examined to confirm the heritability of the mutations. All 36 T1 descendants of M1 were sequenced, 15 of which were wild-type and the others were heterozygous, and they were identical to those found in the T_0_ generation (Figure 1a and b). These findings suggest that mutations caused by the tRNA-processing system are inheritable in *B. rapa*. No off-target mutation was identified at the top two ranking off-targets (Figure S4, see online supplementary material).

### *BraRGL1* mutation accelerates flower bud differentiation and bolting

**Figure 2:**
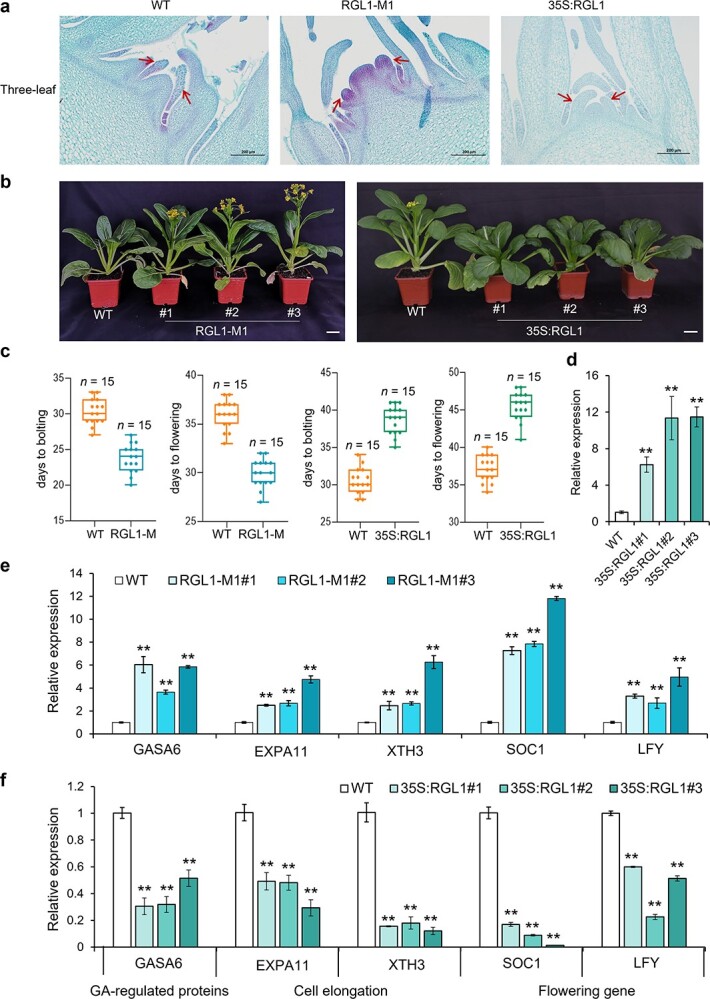
*BraRGL1* negatively regulates the expression of bolting- and flowering-related genes to control bolting. **a** Stem tip longitudinal structures of *BraRGL1-M* knockout lines 35S:*BraRGL1* overexpression lines in the three-true-leaf stag. The red arrow points to the leaf or flower primordium. Scale bar = 200 μm. **b** Phenotypic of the WT, *BraRGL1-M,* and *35S:BraRGL1* lines. Scale bar = 5 cm. **c** Quantification of bolting and flowering time in *BraRGL1-M* and 35S:*BraRGL1* lines. The number of studied accessions for each line is given above the graph. **d** Relative expression of *BraRGL1* in 35S:*BraRGL1* lines. **e** Relative expression of GA-regulated protein (*BraGASA6*), flowering-related genes (*BraSOC1* and *BraLFY*), and expansion-related genes (*BraEXPA11* and *BraXTH3*) in *BraRGL1-M* knockout lines at bolting stage compared to the levels in WT. **f** Relative expression levels of bolting- and flowering-related genes at the bolting stage in the 35S:*BraRGL1* overexpression lines compared with that in WT. Data are presented as the mean ± standard deviation (*n* = 3). Significant deviations from the control determined using Student’s *t*-test (**e** and **f**) (^**^P < 0.01).

We used paraffin sections to investigate the stem tip structure of ‘youlv501’ and ‘youqing 80 day’ two varieties in our previous studies [39]. Neither type differentiated the flower buds completely at the two-true-leaf stage and three-true-leaf stage, but the ‘youlv501’ variety did so at the four-true-leaf stage. Among the six *BraDELLA* genes, only the expression level of *BraRGL1* showed a substantial difference at the two-true-leaf stage, suggesting that *BraRGL1* may be involved in the early bud differentiation of different varieties. To further characterize the biological function of *BraRGL1*, we observed the stem tip structure of *BraRGL1* mutants at the three-true-leaf stage using paraffin sections. WT plants did not initiate flower bud differentiation and were in the leaf primordium stage in the three-true-leaf stage, whereas *BraRGL1* mutants had differentiated flower primordium (Figure 2a). Further statistics on the bolting and flowering phenotypes showed that the flowering time of *BraRGL1* mutants was significantly earlier than that of WT (Figure 2b and c), and the expression levels of GA-regulated gene (*BraGASA6*: *BraA02g023240.3.5C*), flowering-related genes (*BraSOC1*: *BraA05g005290.3.5C*) and (*BraLFY*: *BraA02g045080.3.5C*), expansion gene (*BraEXPA11*: *BraA07g016390.3.5C*), and xyloglucan endotransferase gene (*BraXTH3*: *BraA07g008170.3.5C*) were significantly upregulated in the stem tip of *BraRGL1* mutant (Figure 2e). These findings suggest that the mutation in *BraRGL1* accelerates early flower bud differentiation, thereby encouraging bolting and flowering.

To further validate our experimental results in the *BraRGL1* mutants, we generated *BraRGL1* overexpression lines under the control of the CaMV 35S promoter. The bolting and flowering time of 35S:*BraRGL1* lines were significantly delayed, and the expression levels of *BraRGL1* in the stem tip were significantly increased, while the expression levels of bolting and flowering-related genes were significantly decreased (Figure 2b–d and f). These results further confirmed that *BraRGL1* negatively regulates bolting- and flowering-related genes to control bolting.

### *BraRGL1* loss-of-function mutants are more sensitive to GA signaling

**Figure 3:**
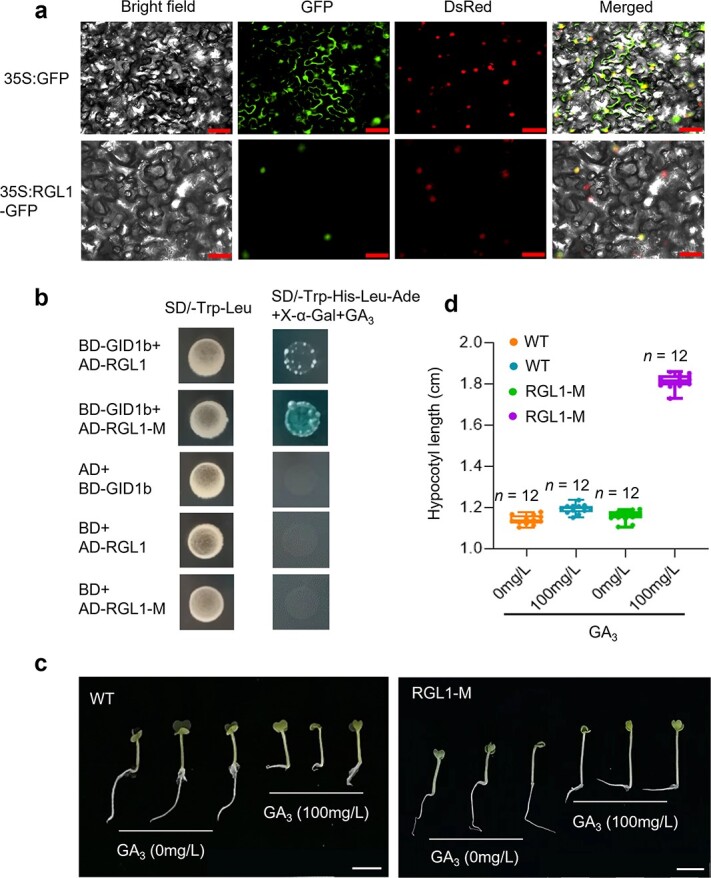
GA_3_ sensitivity of the *BraRGL1* mutants. **a** Subcellular localization of *BraRGL1* in *Nicotiana benthamiana*. DsRed was used to stain the nuclei. Scale bar = 50 μm. **b** Detection of interactions between BraRGL1 proteins and BraGID1b after treatment with 100 mg/L GA_3_. *BraRGL1-M* represents the mutated protein. AD and BD represent empty pGADT7 and pGBKT7, respectively. SD/−Trp-Leu means medium lacked tryptophan and leucine; SD/−Trp-His-Leu-Ade means medium lacked tryptophan, histidine, leucine, and adenine. **c** Sensitivity of *BraRGL1* mutants to GA_3_ determined using a hypocotyl elongation assay with 100 mg/L GA_3_ treatment. Scale bar = 1 cm. **d** Quantification of hypocotyl lengths, as in Figure 2. *n*, number of hypocotyls.

GA signal transduction is negatively regulated by the BraRGL1 proteins and the *BraRGL1* loss of function mutation weakens its inhibitory effect, thereby accelerating the bolting. Subcellular localization analysis revealed that *BraRGL1* was localized in the nucleus (Figure 3a). Expression levels of the GA-regulated protein *BraGASA6* were significantly increased in *BraRGL1* mutants (Figure 2e), suggesting that its loss of function may regulate GA signal transduction. We previously showed that BraRGL1 interacts with BraGIDb in the presence of GA_3_ (200 mg/L) [39]. To further explore whether the *BraRGL1* mutation affects the sensitivity to GA signaling, we determined the degree of interaction BraRGL1 and BraRGL1-M with BraGID1b in the presence of GA_3_ (100 mg/L). BraRGL1 and BraGID1b showed weak interactions, while BraRGL1-M presented strong interactions with BraGID1b (Figure 3b). The addition of a dose gradient of GA_3_ further confirmed the results (Figure S5, see online supplementary material). These findings indicated that *BraRGL1-M* was more sensitive to GA_3_ signaling. The sensitivity of *BraRGL1* mutant to GA signaling was further verified by hypocotyl elongation experiments. Seeds of the WT and *BraRGL1* mutants were sown on seeding medium with or without GA_3_ (100 mg/L). On the third day after sowing, there was no significant difference in the hypocotyl length of WT on the medium with or without GA_3_, whereas the hypocotyl length of *BraRGL1* mutant on GA_3_-containing seeding medium was significantly longer than that of the control (without GA_3_) and WT (Figure 3c and d). These results further confirmed that *BraRGL1-M* mutants were more sensitive to GA_3_ signaling.

### GA_3_ attenuates the interaction between BraRGL1 and BraSOC1

**Figure 4:**
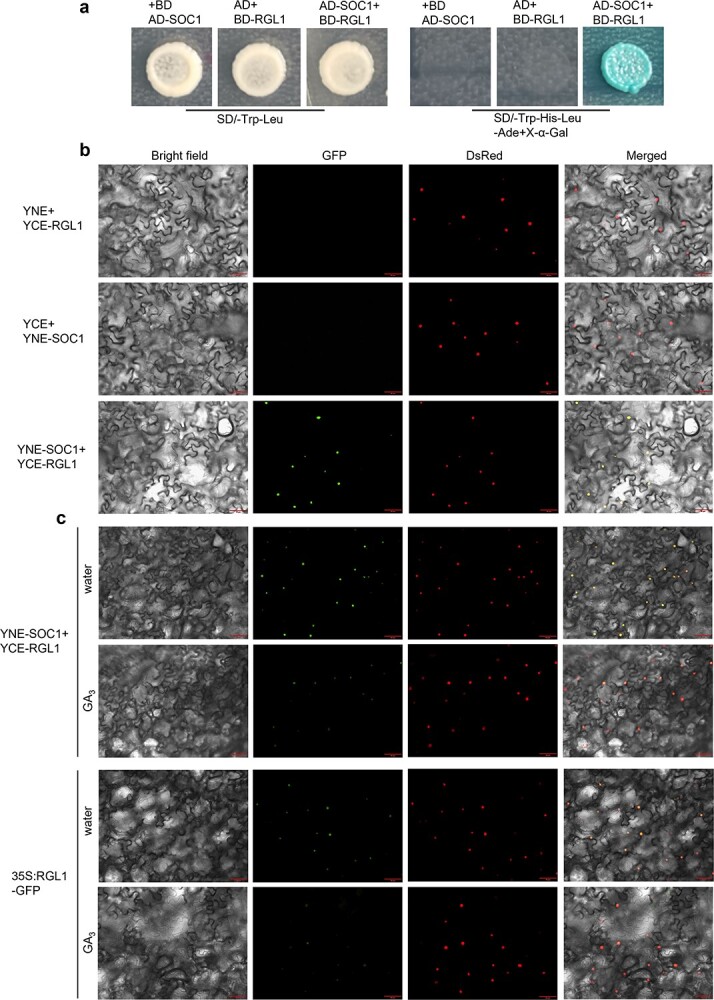
BraRGL1 interacts with BraSOC1. **a** Yeast two-hybrid assay for protein–protein interactions between BraRGL1 and BraSOC1. AD and BD represent empty pGADT7 and pGBKT7, respectively. SD/−Trp-Leu means medium lacked tryptophan and leucine; SD/−Trp-His-Leu-Ade means medium lacked tryptophan, histidine, leucine, and adenine. **b** The interaction between BraRGL1 fusing to C-termini of YFP and BraSOC1 fusing to N-termini of YFP was detected by bimolecular fluorescence complementation assay. **c** Bimolecular fluorescence and 35S: RGL1-GFP fluorescence after GA_3_ treatment. Leaves were sprayed with water or GA_3_ 1 h before observation of the signals. DsRed was used to stain the nuclei. Scale bar = 50 μm.

*BraRGL1-M* mutants exhibit an early bolting and flowering phenotype because they are more sensitive to GA, indicating GA affects bolting and flowering through BraRGL1 proteins, but the underlying molecular mechanism is not clear. DELLA is thought to lack a DNA-binding domain (DBD), indicating that it might play its negative regulatory effects through interacting with other transcription factors [42, 43]. In our previous study, the flowering-promoting factor *BraSOC1* positively regulated bolting and flowering by upregulating *BraEXPA11*, *BraXTH3*, and *BraLFY* upon exogenous GA_3_ treatments [44]. In the present study, BraRGL1 negatively regulated the expression of these genes, thus affecting bolting and flowering. Therefore, we hypothesized that BraRGL1 interacts with BraSOC1 to control bolting. We tested this hypothesis by performing a yeast two-hybrid (Y2H) assay between the BraRGL1 and BraSOC1 proteins. We discovered that BraRGL1 and BraSOC1 interacted to create heterologous dimers (Figure 4a). A bimolecular fluorescence complementation (BiFC) assay was performed *in vivo* to confirm results of the Y2H assay. The association between BraRGL1 and BraSOC1 was validated by GFP fluorescence in the nuclei of plant cells (Figure 4b). We further examined the interaction of BraRGL1 and BraSOC1 after GA_3_ treatment and observed that GA_3_ significantly attenuated the GFP signal (Figure 4c). In addition, GA_3_ administration drastically reduced the 35S: RGL1-GFP fluorescence (Figure 4c). Combined with the signal transduction mechanism of GA_3_ in model plants, we speculate that increased GA_3_ concentration resulted in the degradation of DELLA protein and the release of BraSOC1 from the BraRGL1 and BraSOC1 dimer.

### BraRGL1 inhibits the transcriptional regulation of BraSOC1

**Figure 5:**
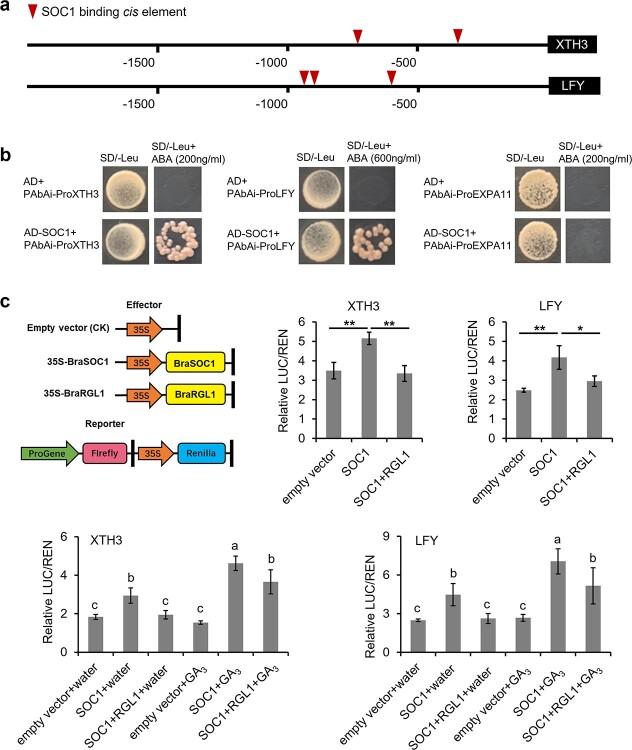
Validation of BraSOC1 and BraRGL1 regulation of *BraXTH3* and *BraLFY*. **a** Location of the *SOC1*-binding elements in the promoter of *BraXTH3* and *BraLFY*. **b** Yeast one-hybrid assays identify the interaction of BraSOC1 with the promoter of *BraXTH3* and *BraLFY*. AD represents empty pGBKT7. SD/−L means medium lacking leucine. **c** Dual luciferase assay to detect BraSOC1, BraRGL1 and their interaction regulate the transcription of *BraXTH3* and *BraLFY*. Empty vector was used as the negative control. Leaves were sprayed with water or GA_3_ 1 h before determination of luciferase activity. Data are presented as the mean ± standard deviation (*n* = 3). Student’s *t* test was used to identify significant differences compared to the control (^*^P < 0.05 and ^**^P < 0.01).

We further identified the target genes regulated by the BraRGL1 and BraSOC1 interactions by analyzing the promoter regions of *BraEXPA11*, *BraXTH3*, and *BraLFY*. The promoter regions of *BraXTH3* and *BraLFY* contained two and three *SOC1*-binding *cis*-elements, respectively, whereas the promoter region of *BraEXPA11* did not (Figure 5a). These elements were located 720 bp and 368 bp upstream of the *BraXTH3* start codon and 959, 928, and 677 bp upstream of the *BraLFY* start codon (Figure 5a). We next performed Y1H assays to assess potential BraSOC1 binding to the target genes promoter. BraSOC1 bound to the both *BraXTH3* and *BraLFY* promoter fragments containing *SOC1*-binding *cis*-elements but did not interact with the *BraEXPA11* promoter (Figure 5b; , Figure S6, see online supplementary material). We further verified their transcriptional regulation of BraRGL1 and BraSOC1 by using a dual luciferase assay. BraSOC1 bound to the promoters of *BraXTH3* and *BraLFY* to induce their transcription, whereas the presence of BraRGL1 inhibited this transcriptional capacity of BraSOC1 (Figure 5c), indicating that BraSOC1 regulates the expression levels of these two genes by interacting with BraRGL1. In addition, GA_3_ enhanced the transcriptional activation capacity of BraSOC1 (Figure 5c), indicating that increased GA_3_ concentrations resulted in the release of BraSOC1 from the BraRGL1 and BraSOC1 dimer, which upregulates the expression of *BraXTH3* and *BraLFY*.

We further examined the interaction between BraRGL1-M and BraSOC1, and the effect of variation in the interaction intensity on downstream gene transcription. Y2H assay showed that the interaction intensity of BraRGL1-M with BraSOC1 is stronger than that of BraRGL1 with BraSOC1 (Figure S7, see online supplementary material). However, DLR assay showed that BraSOC1’s transcriptional activation ability on downstream genes did not change significantly in the presence of BraRGL1-M (Figure S8, see online supplementary material), that is, BraRGL1-M could not inhibit BraSOC1’s transcriptional activation ability on downstream genes, indicating that BraRGL1-M may have lost its inhibitory function. This is consistent with the phenotype of BraRGL1-M mutant with early bolting.

### *BraRGL1-M* mutants advance flower bud differentiation without affecting stalk quality

**Figure 6:**
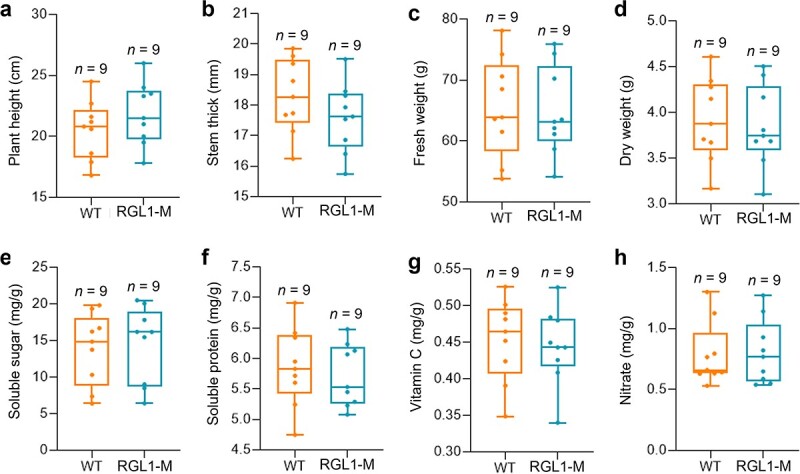
Determination of growth and nutritional indicators of WT and *BraRGL1* mutants. **a** Plant height. **b** Stem thick. **c** Fresh weight. **d** Dry weight. **e** Soluble sugar content. **f** Soluble protein content. **g** Vitamin C content. **h** Nitrate content. *n*, number of plants.

The stalk is not only the main part of the product organ but also the nutrient storage organ in Caixin. Carbohydrates were the main nutrient component in Caixin, and soluble sugars and vitamin C in stalks were significantly higher than those in the leaves [45]. To test whether early flower bud differentiation of *BraRGL1-M* mutants would reduce the quality of stalks, we determined the growth indicators and nutritional indicators in *BraRGL1-M* mutants. The plant height of the *BraRGL1-M* mutants was slightly higher than that of the WT, while the stem diameter was slightly lower than that of the WT, but the difference was not significant (Figure 6a and b), and there were no significant differences in fresh and dry weight between *BraRGL1-M* mutants and WT (Figure 6c and d). In addition, the contents of soluble sugar and nitrate in the *BraRGL1-M* mutants were slightly higher than those in the WT, while soluble protein and vitamin C contents were slightly lower than those in the WT, but the difference was not significant (Figure 6e–h). These results indicated that *BraRGL1-M* mutants promoted flower bud differentiation without affecting the stalk quality, which shortened the growth cycle of Caixin while maintaining yield, providing a scientific basis for further breeding of excellent early maturing varieties.

## Discussion

The CRISPR/Cas9 system has several advantages, enabling almost all genes to be edited; however, the gene targeting efficiency is exhibited in a species-dependent or cell-type-dependent manner [46]. Earlier reports in Fastcycling *B. oleracea DH1012* showed only 10% editing efficiency in the *GA4* gene [19], which is far lower than the mutation efficiency of 91.6% in rice [47], 89% in *Arabidopsis* [48], 87.5% in tobacco [49], 87.5% in petunia [50], and 86.4% in poplars [51]. *BoPDS* and *BoSRK* high-efficiency mutagenesis (68% and 100%, respectively) were achieved using the CRISPR/Cas9 gene editing system based on endogenous tRNA processing in *B. oleracea* [24]. In this study, we achieved efficient inheritable mutagenesis (72.72% and 63.15%) in ‘youlv501’, which was higher than the mutation efficiency of 20%–56% of the three sgRNAs in ‘Youqing 49’ [22]. These results indicate that the CRISPR/Cas9 gene editing system with endogenous tRNA processing is suitable for efficient mutagenesis in *B. rapa*, providing an important technical strategy for gene function identification and functional gene mining of *B. rapa* and other *Brassica* vegetables.

Five DELLA genes have been identified in *Arabidopsis*: *GAI*, *RGA*, *RGL1*, *RGL2*, and *RGL3* [52, 32]. *GAI* and *RGA* inhibit stem elongation and flower development [34, 37]. *RGL2* is a major negative regulator of seed germination [35]. *RGL1* can enhance the roles of *RGA* and *RGL2* in flower development [53]. *RGL3* plays a positive regulatory role in stress resistance [54]. These results suggest that they have redundant and distinct purposes [55, 56]. In this study, flower bud differentiation and bolting time of *BraRGL1* loss-of-function mutants were significantly advanced, and overexpressed plants showed opposite phenotypes, suggesting that *BraRGL1* is a key factor regulating bolting and flowering in *B. rapa*.

A crucial regulating mechanism in the GA signaling pathway is GA-induced DELLA degradation [30, 31]. GID1 acquires the ability to interact with DELLA by binding to active GAs, enabling further interaction with the F box protein. DELLA is polyubiquitinated by E3 ubiquitin ligase SCF^SLY1/GID2^ and is finally degraded by the 26S proteasome. GID2 in rice and SLY1 in *Arabidopsis* bind to DELLA proteins in the presence of GA and promote DELLA protein degradation, thereby activating the GA response [57, 58]. Accordingly, our study showed that BraRGL1 interacted with BraGID1b in the presence of GA_3_. In addition, the interaction between BraRGL1-M and BraGID1b was stronger under the same concentration of GA_3_ treatment, and hypocotyl of the *BraRGL1-M* mutant was significantly extended. This is consistent with the idea that *DELLA* loss-of-function mutants are more sensitive to GA [34–37]. DELLA and TVHYNP domains are critical GA signal sensing domains, but amino acid sequences are not conserved. Therefore, GA-sensitive *BraRGL1-M* mutation may be attributed to the substitution of two amino acids in GA signal suppression region (GRAS domain), which may lead to the loss of DELLA protein inhibitor function [40, 41].

Because DELLA is thought to lack a DBD, intermediate proteins that mediate DELLA/DNA interactions are thought to be required for the activation of DELLA target genes [42, 43]. DELLA and FLC directly interact with each other and probably function in a large complex to repress the target gene expression, thus regulating flowering transition in *Arabidopsis* [59]. In this study, BraRGL1 was found to directly interacts with BraSOC1. *BraSOC1* is crucial for controlling bolting and stem elongation in Caixin [44], indicating that the potential bolting and flowering mechanism of *B. rapa* may be different from that of *Arabidopsis* with particularity and complexity. In addition, GA induces the rapid degradation of DELLA proteins by 26S proteasome, resulting in a reduction in the interaction strength of the protein dimers [43, 60]. The bimolecular fluorescence complementation assay when imposing GA_3_ supported the idea that increased GA levels promoted the GID1 receptor-mediated ubiquitination degradation of DELLA proteins, thereby releasing BraSOC1. Therefore, the BraRGL1-BraSOC1 module regulates the bolting and flowering by modulating the GA signal transduction pathway.

**Figure 7:**
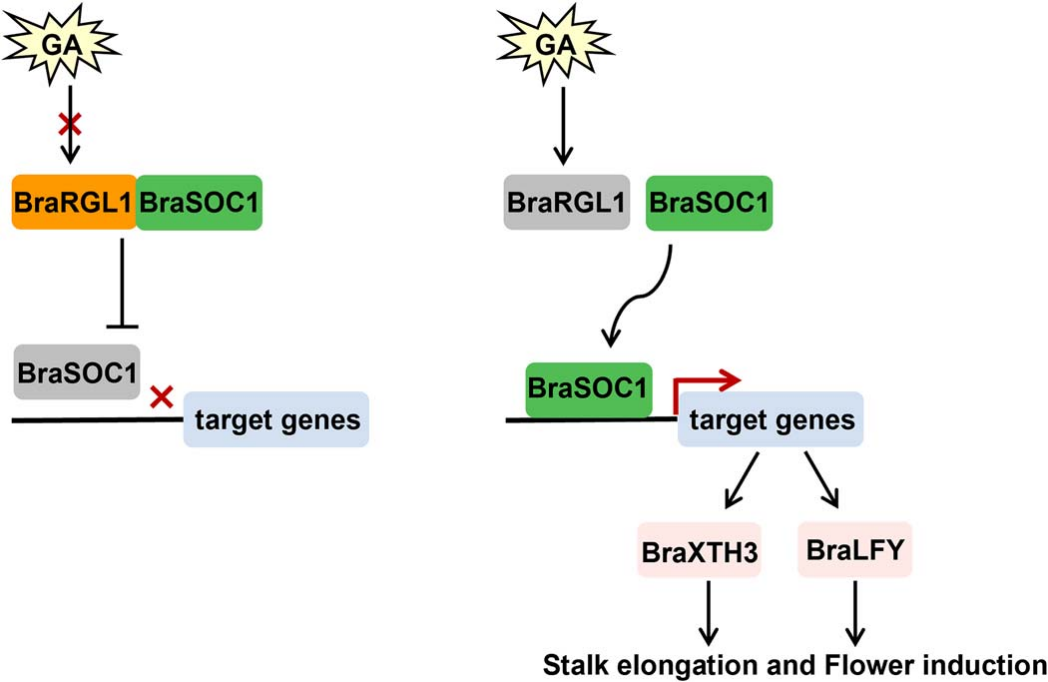
Schematic model of the BraRGL1-mediated GA pathway regulating bolting and flowering in *Brassica rapa*. Increased GA_3_ concentration results in BraRGL1 degradation and the release of BraSOC1 from the BraRGL1 and BraSOC1 dimer, which upregulates the expression of *BraXTH3* and *BraLFY*, thus promoting bolting and flowering.

Increased cell expansion may be the cause of stalk elongation of Caixin [44]. *BraEXPA11* and *BraXTH3* are the key factors involved in cell expansion [61, 62]. *EXPA11* enabling cell wall expansion by reducing the viscosity of polysaccharides between cell walls [63]. *XTH3* regulates cell wall relaxation through cleaving xyloglucan chains [64]. GA_3_ treatment decreased the expression of the *BraRGL1* genes and increased the expression of *BraSOC1*, *BraGASA4*, *BraEXPA11*, and *BraXTH3* [39, 44]. GA stimulates the expression of *EXPA* and *XTH* to encourage cell wall relaxation and elongation [65]. *LFY* is a flowering factor that acts downstream of *SOC1* in *Arabidopsis* [66]. In this study, in addition to *BraLFY*, we also identified another target gene of *BraSOC1*, *BraXTH3*, which is closely related to the function of *BraSOC1* in regulating stem elongation [44]. We determined that BraSOC1 binds directly to the *BraXTH3* and *BraLFY* promoters. Although *BraEXPA11* showed a consistent expression pattern with *BraSOC1*, *BraXTH3*, and *BraLFY* in *BraRGL1* mutants and overexpressed plants, there was no *SOC1* binding site, suggesting indirect regulation. However, this conclusion needs to be confirmed by further experiments, as we did not examine other copies of BraEXPA11. DELLA interacts with PIF3 and PIF4 and blocks their DNA-binding, whereas GA-induced DELLA degradation promotes the activation of PIF3 and PIF4 to target genes, thus promoting *Arabidopsis* hypocotyl cell extension [43, 67]. In this study, the presence of BraRGL1 inhibited the activation ability of *BraSOC1* for *BraXTH3* and *BraLFY* transcription, whereas GA_3_ enhanced the activation ability of *BraSOC1*, suggesting that the BraRGL1-BraSOC1 module regulates the bolting and flowering through controlling the expression of *BraXTH3* and *BraLFY*. Based on these findings, we hypothesized that the increase of GA_3_ content causes the degradation of BraRGL1 protein, which releases *BraSOC1*, thus upregulating the expression of *BraXTH3* and *BraLFY* genes, finally promoting bolting and flowering in *B. rapa* (Figure 7).

There have been many studies on GA-induced ubiquitination degradation of DELLA protein, but the specific mechanism of DELLA protein action remains unclear, such as how DELLA protein interacts with downstream genes and how it inhibits transcriptional activity of downstream genes. In this study, BraRGL1-M had stronger interaction with BraSOC1, while BraRGL1-M did not inhibit the transcriptional activation of BraSOC1 (Figs ure S9 and ure S10, see online supplementary material). This indicated that BraRGL1-M only lost its inhibitory function but did not lose the ability of protein interaction. In addition, *rga-1* and *sln1c* accumulate mutated DELLA proteins, but they also lack repressive function [68]. These results indicated that the inhibition effect of DELLA protein on downstream genes may not be realized through simple protein accumulation or interaction. In addition, it was reported that the inhibition effect of DELLA protein on downstream genes may be related to protein phosphorylation and ubiquitination [60], which also indicated the complexity of the mechanism of DELLA protein and interacting proteins and that is what we will tackle next.

Early flower bud differentiation can significantly shorten the growth cycle of *B. rapa*, which is of great economic significance. In this study, although flower bud differentiation was advanced in *BraRGL1-M* mutants, growth and nutrition indices were not affected (Figure 6). This indicates that *BraRGL1-M* mutants may have applications in breeding. The gene *TaDEP1* that controls inflorescence formation, spike grain growth, and grain yield of wheat was knocked out using CRISPR/Cas9 technology to shorten inflorescence internode length, increase grain number per spike, and increase grain yield [69]. Shi *et al.* obtained maize varieties with high yield and drought tolerance by knocking out the *ARGOS*, which regulates ethylene biosynthesis and signal transduction [70]. Using the CRISPR/Cas9 system to knock out the tomato flowering inhibitory gene *SP5G* can quicken tomato blossoming and enhance the compact limited growth habit, leading to rapid fruit ripening [71]. In addition, the stability and inheritance of mutations are crucial for the generation of mutants using the CRISPR/Cas9 system [13, 19]. In the present study, the CRISPR/Cas9 system-induced genomic mutations in *B. rapa* were stable and inheritable, which laid the foundation for further segregation of excellent early maturing varieties.

In summary, we demonstrated that the CRISPR/Cas9 system based on endogenous tRNA processing is an effective tool for studying gene function in *B. rapa*, achieving high efficiency and inheritable mutagenesis of multiple targets. *BraRGL1* plays a crucial biological role in the early flower bud differentiation of *B. rapa*. BraRGL1 and BraSOC1 interact to regulate the expression of *BraXTH3* and *BraLFY*, thereby controlling the bolting and flowering. These findings expand our understanding of the regulatory mechanisms that underlie bolting and flowering in *B. rapa*. In addition, *BraRGL1-M* mutant promoted flower bud differentiation without affecting the stalk quality, which provides a scientific basis for the further application of breeding strategies to control this important trait.

## Materials and methods

### Vector construction

The target sites of *BraPDS* and *BraRGL1* were designed as described by CRISPR-GE (http://skl.scau.edu.cn/) [72]. Four optimal target sites were selected according to sequence, position, positive and negative strands, GC content, potential off-target sites, and valuation information of the candidate target sites. Complementary oligos of the target sequences were synthesized, and double-stranded DNA fragments were formed after denaturation (95°C for 5 min) and renaturation (room temperature for 1 h). Four target sites were then cloned into four sites (*Bbs*I, *Bsa*I, *Bsm*BI, and *Bfu*AI) of the tRNA-sgRNA vectors to generate At6–26::tRNA-sgRNA-*BraPDS*-1234 and At6–26::tRNA-sgRNA-*BraRGL1*–1234 expressing cassettes. Finally, gene editing vectors of *BraPDS* and *BraRGL1*, respectively, were created by cloning the expressing cassettes into the *Bam*HI and *Eco*RI sites of the pCACas9 vector (Figure S9, see online supplementary material).

### *Agrobacterium*-mediated transformation in *B. rapa*

Caixin (*B. rapa* ssp. *Chinensis* var. *parachinensis*) is a varietas of pak choi originally from South China. A highly inbred line ‘youlv501’ from our laboratory was used in this study for genetic transformation. Caixin was subjected to cotyledon transformation using a previously reported procedure [44] (Figure S10, see online supplementary material). Briefly, three days after emergence, the cotyledons of sterile seedlings were removed. The cotyledon explants were subsequently pre-cultured medium to initiate callus growth for 3 days. The preincubated cotyledons were then transferred to the co-cultivation medium in the dark for 3 days after being infected with liquid medium containing *Agrobacterium* (GV3101 strain containing corresponding plasmids, OD600 = 0.6) for 10 min. After inhibition of *Agrobacterium* for 7 days, the explants were placed in selection medium [co-cultivation medium with 4 mg/L phosphinothricin (PPT) or 10 mg/L Kanamycin (Kan)]. The PPT-resistant shoots were placed in the rooting medium upon reaching a height of 2–3 cm.

### Mutation detection

Genomic DNA was extracted from the leaves of individual PPT-resistant plants. DNA from the positive transgenic plants served as the template for PCR amplification using gene-specific primers, and the PCR products were directly sequenced for indel detection. PCR amplicons with double peaks were ligated into the pMD19-T vector, and eight monoclones were randomly selected for further sequencing to determine the mutation pattern.

### Off-target evaluation

Potential off-target sites were predicted using the CRISPR-GE system (http://skl.scau.edu.cn/) [72]. Two potential off-target sites with the highest off-target risk were selected for further confirmation, and they contained less than or equal to 4-bp mismatches in the 12-bp seed sequence with the target sites. Using gene-specific primers, three putative off-target regions were cloned and the PCR results were examined using Sanger sequencing. DNAMAN software was used to examine sequencing outcomes.

### The generation and identification of 35S: *BraRGL1* lines

To create the 35S: *BraRGL1* overexpression vector, the full-length CDS of *BraRGL1* without stop codons was cloned into the *Xba*I and *BamH*I sites of the pBI121-GFP vector. The resulting construct was introduced into the GV3101 strain of *Agrobacterium*, which subsequently used the cotyledon method to convert it into Caixin. Genomic DNA was extracted from the Kan-resistant plants using the CTAB method. Specific primers were used to amplify the target gene, and the resulting PCR products were sequenced and aligned.

### Quantitative reverse transcription PCR (qRT–PCR) analysis

Total RNA was extracted from the stem tips of WT and *BraRGL1* transgenic plants, and qRT–PCR analyses were carried out using ChamQ SYBR Color qPCR Master Mix under the following PCR conditions: 5 min at 95°C, 40 cycles of 10 s at 95°C, 30 s at 55°C, 20 s at 72°C. The internal reference gene for gene expression analysis was glyceraldehyde-3-phosphate dehydrogenase (*GAPDH*).

### Subcellular localization

Full-length coding sequences without stop codons of *BraRGL1* were cloned into the *Age*I site of the pEAQ-EGFP vector and fused with green fluorescent protein (GFP). Young tobacco leaves were invaded by the GV3101 strain for 2 days, which included nuclear localization signal (NLS-DsRed) and the necessary constructs. A laser-scanning confocal microscope was used to detect GFP fluorescence at 448 nm. At 550 nm, DsRed was observed to represent the nucleus.

### Histological analysis of the *BraRGL1* mutants

The stem tips (5 mm) of WT and *BraRGL1* mutant plants were collected and immersed in FAA fixative solution (70% alcohol: acetic acid: formaldehyde = 90:5:5) for 20 min before being vacuum pumped and incubated at 4°C. The materials were dehydrated in 70% alcohol for two days before being embedded in paraffin. The slices were then stained with a reddish-green dye so that the cell structure could be seen.

### Yeast two-hybrid assay (Y2H)

A combination of stem and leaf cDNA from WT and *BraRGL1* mutant plants was used to amplify the full-length coding sequences of *BraRGL1*, *BraRGL1* mutant gene (*BraRGL1-M*), and *BraGID1b*. To create yeast two-hybrid vectors, the full-length CDSs of *BraRGL1* and *BraRGL1-M* were cloned into *Eco*RI and *Bam*HI sites of pGADT7 vector, and the full-length CDS of *BraGID1b* were cloned into *Eco*RI and *Bam*HI sites of pGBKT7 vector. Yeast strain Y2Hgold was transformed with the recombinant vector to produce fusion proteins. Diploids were selected on SD/−Trp-Leu medium and interactions were validated on SD/−Trp-Leu-His-Ade medium with X-a-Gal. The sensitivity of BraRGL1 and BraRGL1-M to GA_3_ was validated by the interaction of BraRGL1 and BraRGL1-M with BraGID1b in the presence and absence of GA_3_.

### Hypocotyl elongation assay

The sterile seeds of WT and *BraRGL1* mutants were seeded on seeding medium with or without 0.3 mM GA_3_ (MS medium containing 1/2 MS, 1% sucrose and 0.6% agar [pH 5.8]). On the third day after sowing, the hypocotyl elongation was observed and photographed. Hypocotyl length was measured using Image J software. According to the hypocotyl elongation of the WT and *BraRGL1* mutants, the sensitivity of *BraRGL1* mutants to GA_3_ was verified.

### Bimolecular fluorescence complementation (BiFC) assay

To create fusion proteins, the full-length CDSs of BraRGL1 and BraSOC1 without stop codons were cloned into *Bam*HI and *Sal*I sites at the N- or C-termini of the pSPYNE-35S and pSPYCE-35S vectors, respectively. The recombinant vectors were transferred into the *Agrobacterium* strain GV3101. Young tobacco leaves also harbored the *Agrobacterium* strain carrying DsRed and the recombinant plasmid. DsRed protein and GFP fluorescence were observed at 550 and 448 nm, respectively, after two days of incubation.

### Yeast one-hybrid assay (Y1H)

Full-length CDS of *BraSOC1* (*BraA05g005290.3.5C*) was cloned into pGADT7 as a prey vector. The promoter fragments containing the *SOC1*-binding *cis*-elements of the target genes (*BraA07g016390.3.5C*, *BraEXPA11*; *BraA07g008170.3.5C*, *BraXTH3*, and *BraA02g045080.3.5C*, *BraLFY*) were individually cloned into *Hind*III and *Kpn*I sites of the pAbAi bait vectors. The linearized pAbAi constructs were transformed into the Y1H Gold yeast strain and incubated on SD/−Trp medium at 30°C for three days. Positive clones were collected and inoculated on the SD medium lacking Leu (SD/−Leu), with or without aureobasidin A at the selected concentration. After 2–3 days, the binding activity of *BraSOC1* to the target genes was evaluated.

### Dual-luciferase assay (DLR)

Full-length coding sequences of *BraRGL1* and *BraSOC1* were cloned into *Bam*HI and *Eco*RI sites of pGreenII 62-SK vector. The promoter fragments containing the *SOC1*-binding *cis*-elements of *BraEXPA11*, *BraXTH3,* and *BraLFY* were cloned into *Kpn*I and *Nco*I sites of pGreenII0800-LUC vector, respectively. Young tobacco leaves were infiltrated with the effector and reporter of the GV3101 *Agrobacterium* strain for 3 days. Firefly LUC and Renilla LUC (REN) activity was measured using the Dual-Luciferase® Reporter Assay System (Promega, Madison, USA).

### Growth and phytochemical determination

The plants were harvested 37–39 days after sowing, and fresh and dry weights (1 h at 105°C and 48 h at 75°C before determination.) were measured (nine biological replicates per treatment). A ruler was used to measure plant height (cm), a Vernier caliper was used to measure stem thickness (mm), and an electronic balance was used to measure dry and fresh weights.

Distilled water (10 mL) and fresh frozen samples (0.5 g) were incubated in a boiling water bath for 30 min. Then, 5 mL of vitriol, 0.5 mL of anthrone ethyl acetate and 1.9 mL of distilled water were combined with 0.1 mL of the supernatant. After cooling, the soluble sugar content was measured using a UV spectrophotometer at 630 nm [73].

Fresh frozen tissue (0.2 g) was added to 5 mL of distilled water. The supernatant was centrifuged, then diluted with the same amount of distilled water before adding 4 mL of Coomassie brilliant blue G-250 solution. The soluble protein content was measured using a UV spectrophotometer at 595 nm [74].

Fresh frozen samples (0.5 g) were crushed into pulp with 1 mL of 15% potassium ferrocyanide, 1 mL of 30% zinc sulfate, and 3 mL of 1% oxalic acid. Phosphate-acetic acid (1 mL), 5% vitriol (2 mL), and ammonium molybdate (4 mL) were combined with 10 mL of extraction solution. After 15 min, vitamin C content was determined at 500 nm using a UV–visible spectrophotometer [75].

Freshly frozen tissue (0.2 g) was boiled for 30 minutes after soaking in 10 mL of distilled water. After the extract was filtered, 0.1 mL of the extraction solution containing 0.1 mL of salicylic and sulfuric acid (5%) and 9.5 mL of NaOH (8%) was added. A UV spectrophotometer was used to detect the nitrate content at 410 nm [76].

## Supplementary Material

Web_Material_uhad119

## Data Availability

All data generated or analysed during this study are included in this published article and its supplementary information files.
