# Supplementary material for: Role of BraRGL1 in regulation of Brassica rapa bolting and flowering
Source: Hortic Res. 2023 Jun 2;10(8):uhad119. doi: 10.1093/hr/uhad119 (PMC10402658; doi:10.1093/hr/uhad119)
Supplement: Web_Material_uhad119 [file web_material_uhad119.docx]

**Supplementary Figures**

**
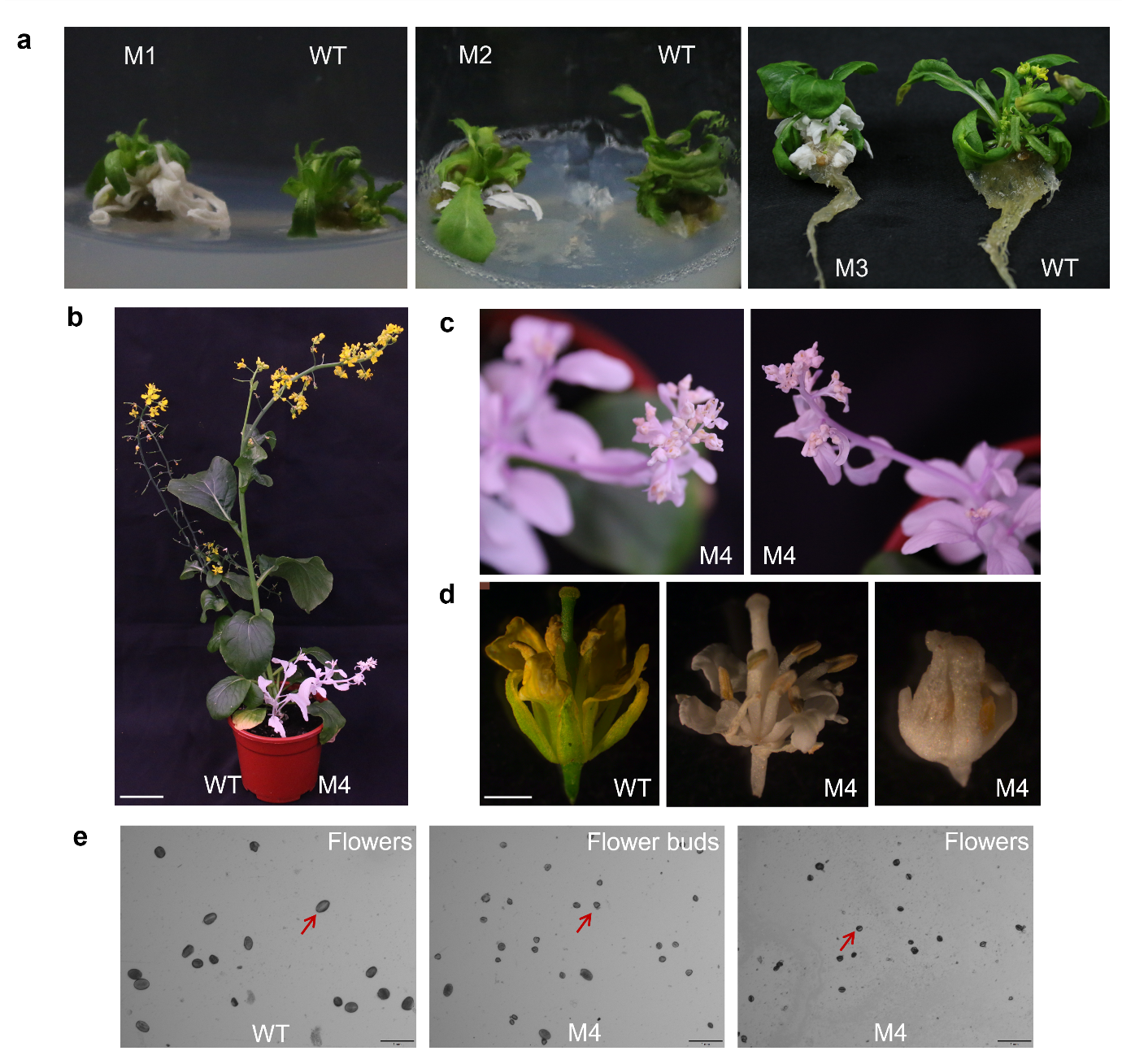
**

**Fig. S1** **Albino phenotype of the *BraPDS* mutants. a, b** Comparison chart of wild type (WT) and four *BraPDS*-mutated plants (M1, M2, M3, and M4). Scale bar = 5 cm. **c** Albino phenotype of the M4 mutant. **d** Phenotypes of WT and M4 mutant flowers. Scale bar = 2 mm. **e** Pollen development of the WT and M4 mutant. The red arrow points to the pollen. Scale bar = 1 mm.

**
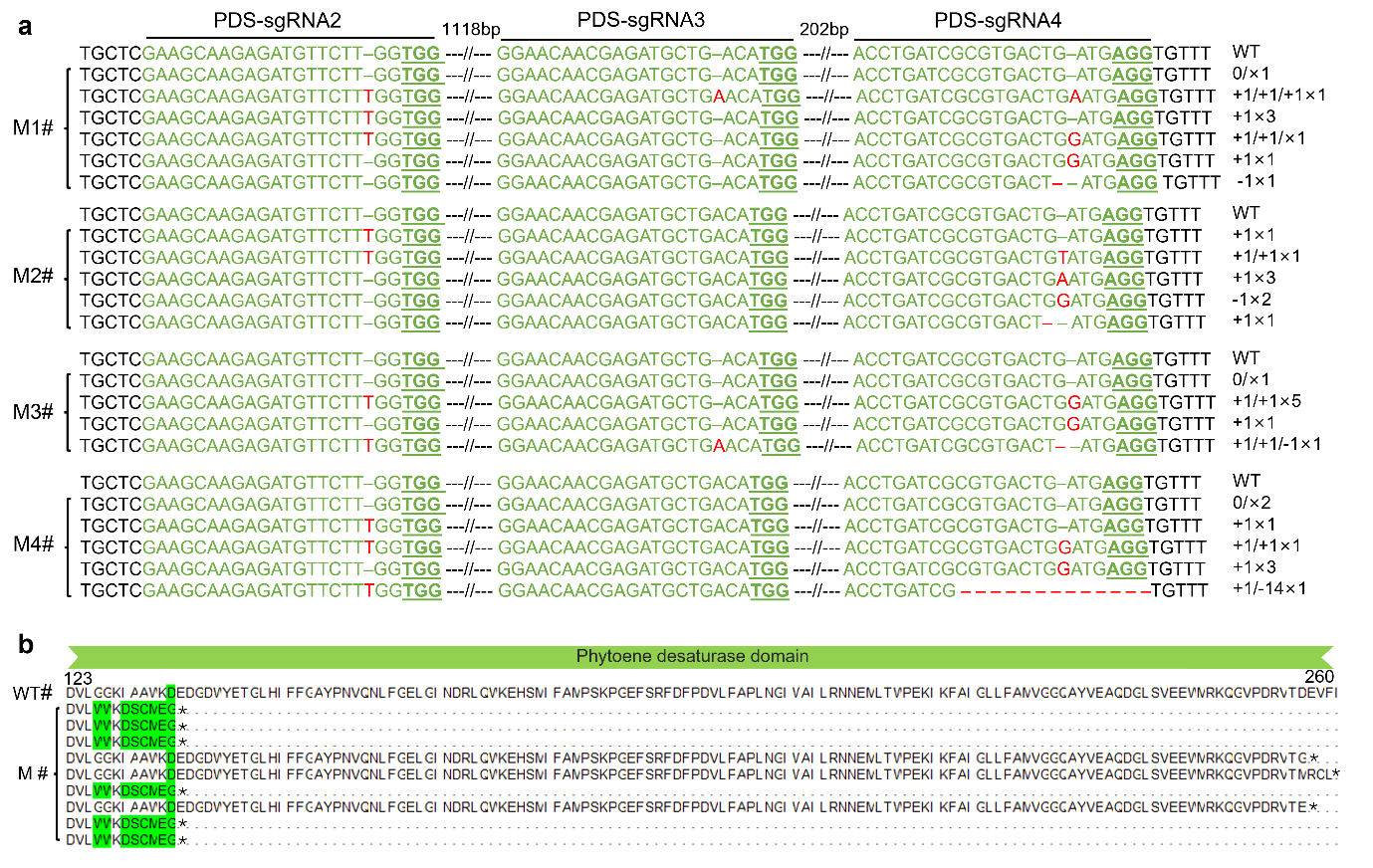
**

**Fig. S2 Mutation type of *BraPDS*. a** Nucleotide sequence alignment of target sites among WT and four mutants (M1, M2, M3, and M4). The PAM sequence is underlined. The target sequences are shown in green with mutations in red. **b** Protein sequence alignment of the nine mutation types. Amino acid sequences 123 through 260 are shown. Mutant amino acids are highlighted in green shading. * indicates terminator.

**
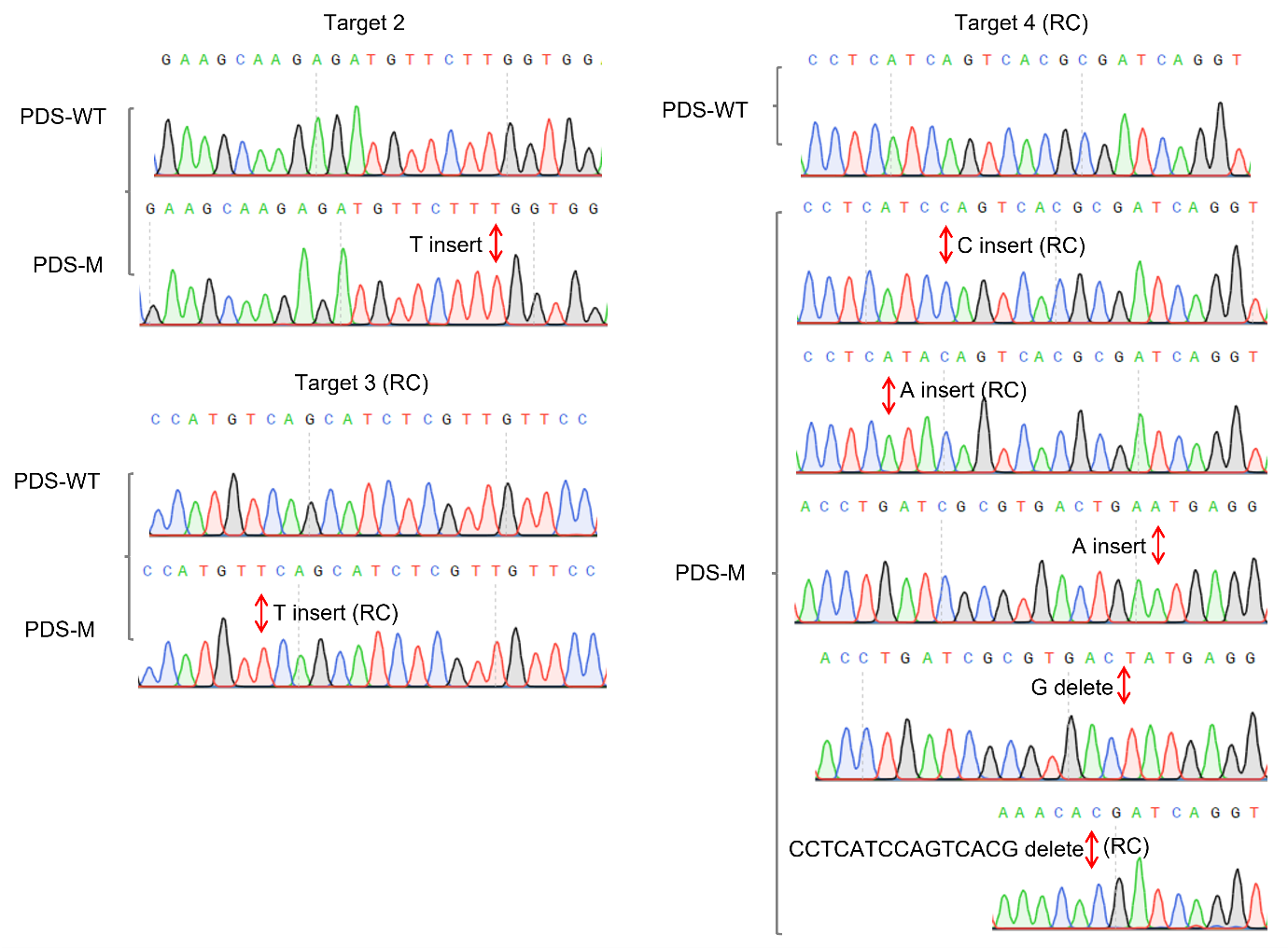
**

**Fig. S3** Chromatogram of different mutation types at target sites in *BraPDS*. RC, reverse complementary strand. The red arrow points to the mutation site.

**
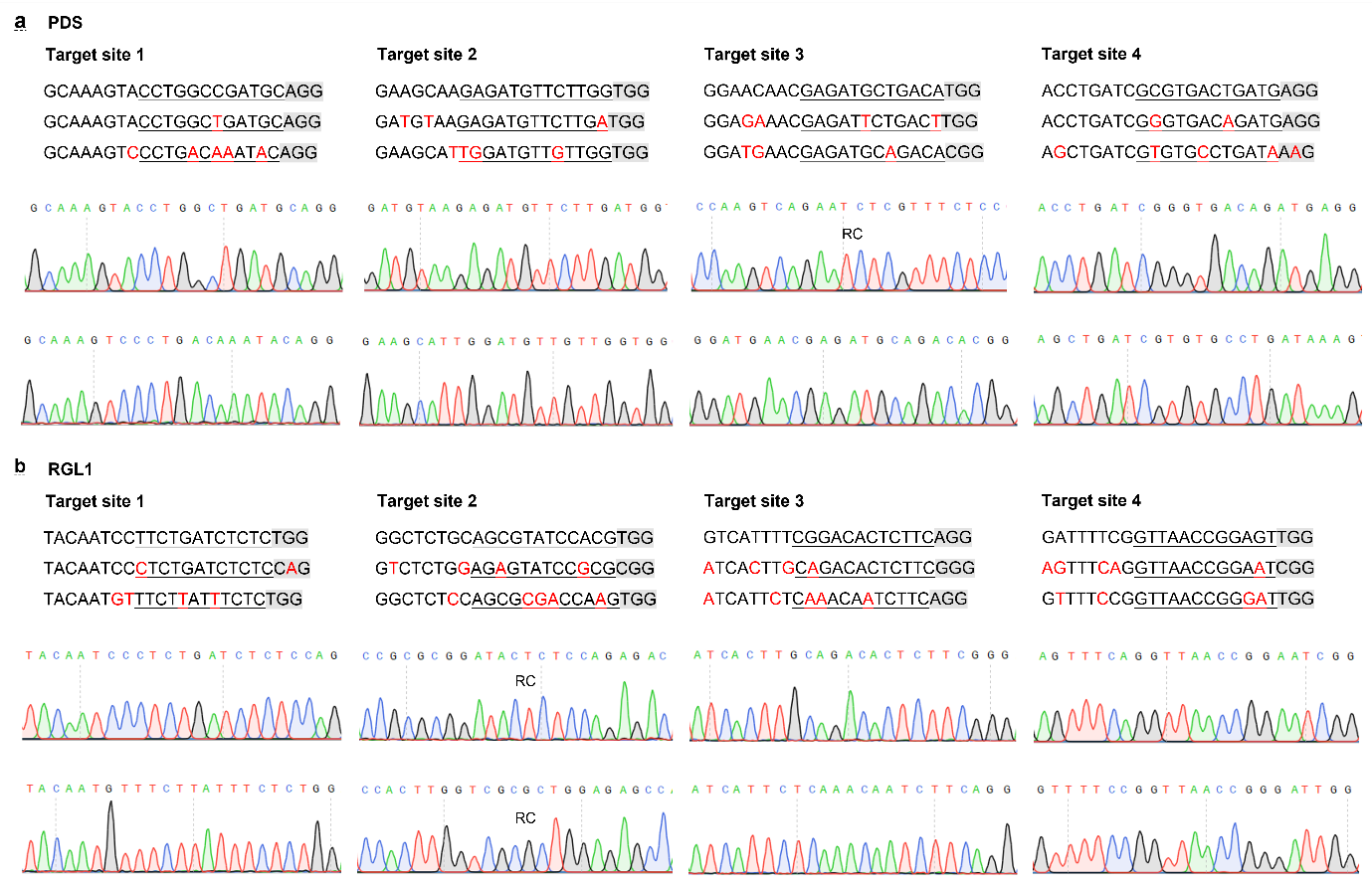
**

**Fig. S4** Potential off-target analysis of *BraPDS* (**a**) and *BraRGL1* (**b**) genes. The upper row is the target sequence, the lower row is the potential off-target sequence. The seed sequence is underlined. PAM is highlighted in gray. Below are the sequencing peaks of the off-target sites. RC, reverse complementary strand.

**
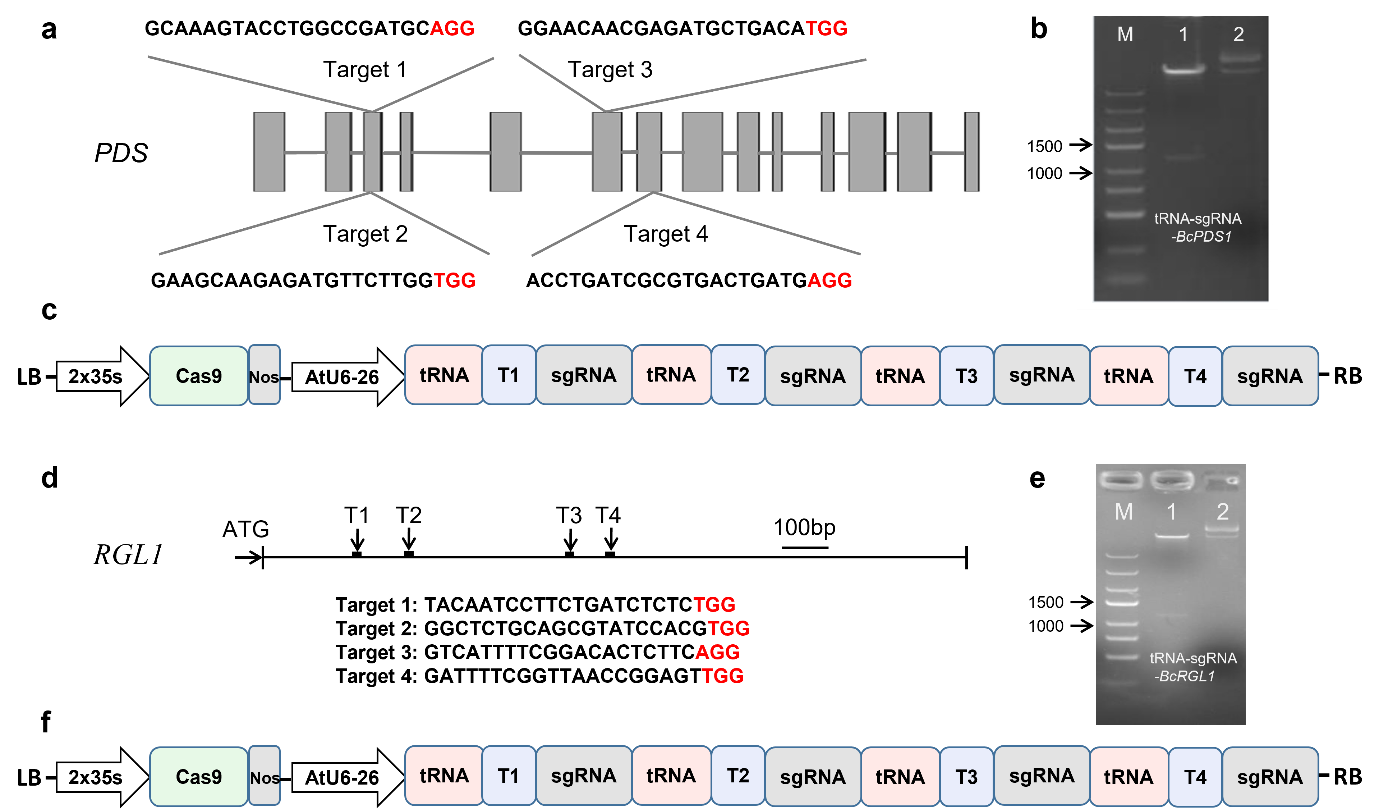
**

**Fig. S5 Construction schema of *BraPDS* and *BraRGL1* editing vectors. a, d** Schematic model of *BraPDS* and *BraRGL1* genes fragments with the indicated four target sites. In panel a, gray boxes indicate exons, and the black lines indicate introns. In panel b, *BraRGL1* has no introns, and the black lines indicate exons. The target sequence is displayed in black letters. The PAM sequence (NGG) is highlighted in red. **b, e** Gel electrophoresis of the cloned fragment of the tRNA-sgRNA expression cassette. Lane 1, Plasmid digested by PvuI; lane 2, plasmid DNA. **c, f** Diagram showing the modified CRISPR/Cas9 vector based on the tRNA-processing system with multiplex sgRNAs for the *BraPDS* and *BraRGL1* genes.

**
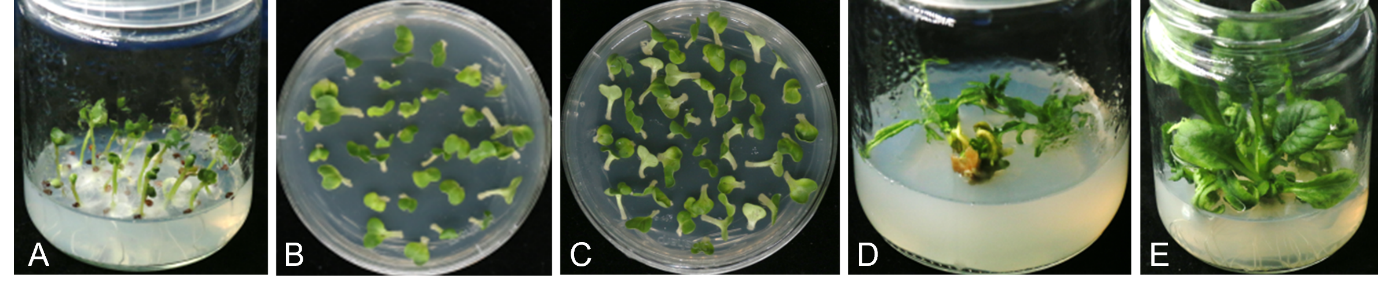
**

**Fig. S6** *Agrobacterium*-mediated transformation in *B. campestris*. (**a**) Septic seedling culture; (**b**) Pre-culture; (**c**) co-culture; (**d**) resistance screening; (**e**) generated roots.

**
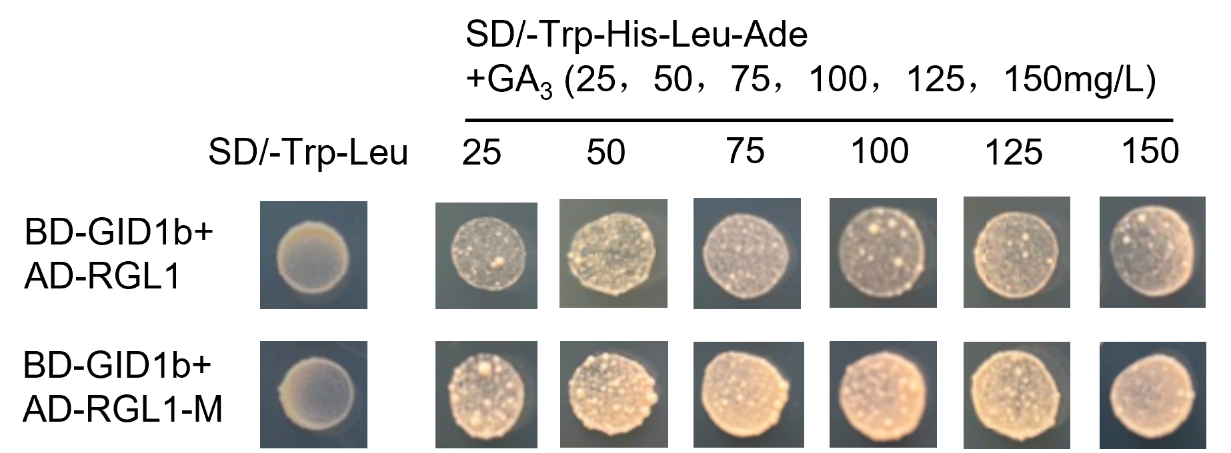
**

**Fig. S7** Detection of interactions between BraRGL1 and BraGID1b proteins after the addition of a dose gradient of GA_3_. *BraRGL1-M* represents the mutated protein. AD and BD represent empty pGADT7 and pGBKT7, respectively. SD/‐Trp‐Leu means medium lacked tryptophan and leucine; SD/‐Trp‐His‐Leu‐Ade means medium lacked tryptophan, histidine, leucine, and adenine.


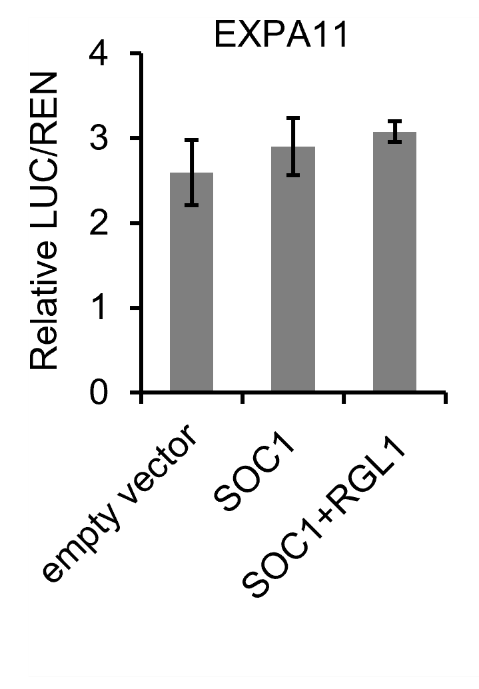


**Fig. S8** Dual luciferase assay to detect BraSOC1, BraRGL1 and their interaction regulate the transcription of *BraEXPA11*. Empty vector was used as the negative control. Data are presented as the mean ± standard deviation (n = 3). Student’s t-test was used to identify significant differences compared to the control (*, P < 0.05 and **, P < 0.01).


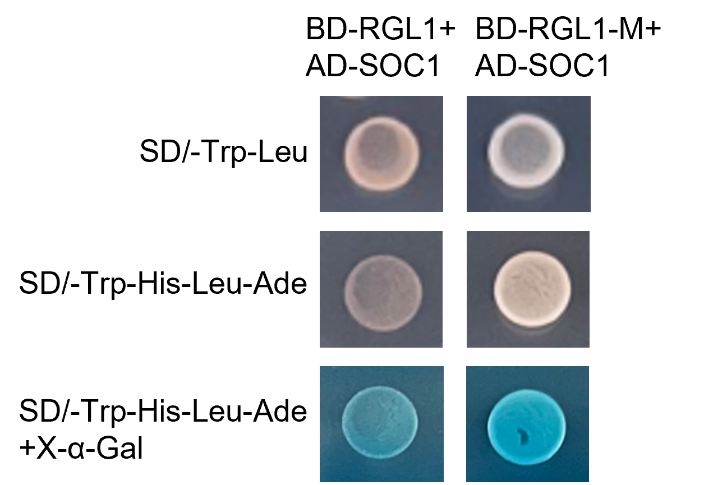


**Fig. S9** Detection of interactions between BraRGL1-M and BraSOC1 proteins. AD and BD represent empty pGADT7 and pGBKT7, respectively. SD/‐Trp‐Leu means medium lacked tryptophan and leucine; SD/‐Trp‐His‐Leu‐Ade means medium lacked tryptophan, histidine, leucine, and adenine. Positive bacteria were stained with X‐α‐Gal.


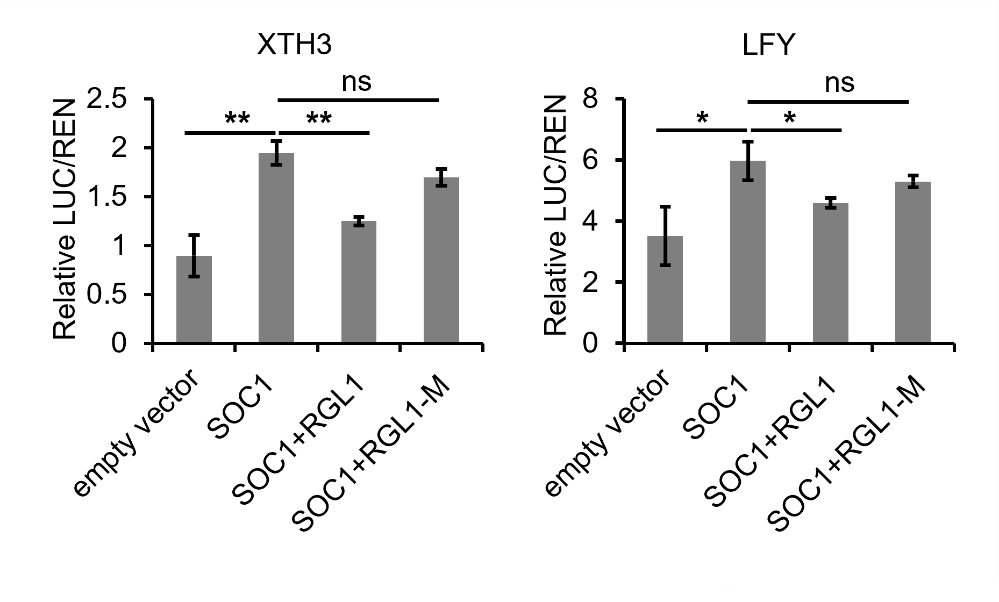


**Fig. S10** Dual luciferase assay to detect BraSOC1, BraRGL1-M and their interaction regulate the transcription of *BraXTH3* and *BraLFY*. Empty vector was used as the negative control. Data are presented as the mean ± standard deviation (n = 3). Student’s t-test was used to identify significant differences compared to the control (*, P < 0.05 and **, P < 0.01).

**Supplementary Tables**

**Table S1.** Mutagenesis frequencies in the T0 generation lines and different sites of the *BraPDS* and *BraRGL1* genes.

| Target genes | Number of  plants analysed | Number of  mutated lines | Mutation  efficiency  (%) | Site1 Mutation  frequency  (%) | Site2 Mutation  frequency  (%) | Site3 Mutation  frequency  (%) | Site4 Mutation  frequency  (%) |
| --- | --- | --- | --- | --- | --- | --- | --- |
| BraPDS | 22 | 16 | 72.72 | 0 | 50.0 | 6.25 | 71.88 |
| BraRGL1 | 19 | 12 | 63.15 | 31.58 | 0 | 63.15 | 57.89 |

**Table S2.** Primers used in this study.

| **Gene** | **Primer name** | **Sequence** (5′-3′) |
| --- | --- | --- |
| BraPDS | BraPDS-CDS-F | ATGGTTGTGTTTGGGAATGTTT |
|  | BraPDS-CDS-R | CTAATCAACAGACCGGAACTGC |
|  | Off-target1-F1 | GGAAGCTGCAAGTTTGTCTGCA |
|  | Off-target1-R1 | CCTAAGATCTGTAACACTCTAT |
|  | Off-target1-F2 | GATGTGAGGATACCATATCAGC |
|  | Off-target1-R2 | TTGGGGACAATTCTAGCTCTGT |
|  | Off-target2-F1 | CACATCTGGAAAGAACTGGAGA |
|  | Off-target2-R1 | TCATTTCGACATATCAATAAAA |
|  | Off-target2-F2 | GTAGAGAACACAGGAGCCGTCG |
|  | Off-target2-R2 | CTTAATAAGGTTAATAGAGACA |
|  | Off-target3-F1 | ATGGCGGCGAGCATCGGAATCA |
|  | Off-target3-R1 | AACACCAACAGTGAATCAACGG |
|  | Off-target3-F2 | CCCAAGAATCCGTTAGAAAAGA |
|  | Off-target3-R2 | CCATATATGTTCGACCAAATCT |
|  | Off-target4-F1 | TGTTGAGGCTCAAGATGGTTTA |
|  | Off-target4-R1 | AGAGTAATAGAGAGAGAGAGGA |
|  | Off-target4-F2 | CCGCCGACCCCAGAAGAAACCC |
|  | Off-target4-R2 | TTTGGTCTGAACAGCAATCTTT |
| BraRGL1 | BraRGL1-CDS-F | ATGAAGAGAGAGCATGGTCACCG |
|  | BraRGL1-CDS-R | TTATTCCACACGATTGAAACGCC |
|  | BraRGL1-RT-F | GATACTGTTCATTACAATCCTT |
|  | BraRGL1-RT-R | AACGAGCACGTCAGCTAGCTTC |
|  | BraRGL1-GFP-F | ctgcccaaattcgcgaccggtATGAAGAGAGAGCATGGTCACCG |
|  | BraRGL1-GFP-R | gcccttgctcaccataccggtTTCCACACGATTGAAACGCC |
|  | 35S:BraRGL1-GFP-F | CCACTGACGTAAGGGATGACGC |
|  | 35S:BraRGL1-GFP-R | CATCGCAAGACCGGCAACAG |
|  | AD-BraRGL1-F | gccatggaggccagtgaattcATGAAGAGAGAGCATGGTCACCG |
|  | AD-BraRGL1-R | cagctcgagctcgatggatccTTATTCCACACGATTGAAACGC |
|  | BD-BraRGL1-F | atggccatggaggccgaattcATGAAGAGAGAGCATGGTCACCG |
|  | BD-BraRGL1-R | ccgctgcaggtcgacggatccTTATTCCACACGATTGAAACGC |
|  | YCE-BraRGL1-F | cgcgccactagtggatccATGAAGAGAGAGCATGGTCACCG |
|  | YCE-BraRGL1-R | ggtaccctcgaggtcgacTTATTCCACACGATTGAAACGC |
|  | pGreenII 62-SK-BraRGL1-F | cgctctagaactagtggatccATGAAGAGAGAGCATGGTCACCG |
|  | pGreenII 62-SK-BraRGL1-R | gataagcttgatatcgaattcTTATTCCACACGATTGAAACGC |
|  | AD-BraRGL1-M-F | gccatggaggccagtgaattcATGAAGAGAGAGCATGGTCACCG |
|  | AD-BraRGL1-M -R | cagctcgagctcgatggatccTTATTCCACACGATTGAAACGC |
|  | BD-BraRGL1-M -F | atggccatggaggccgaattcATGAAGAGAGAGCATGGTCACCG |
|  | BD-BraRGL1-M -R | ccgctgcaggtcgacggatccTTATTCCACACGATTGAAACGC |
|  | pGreenII 62-SK-BraRGL1-M-F | cgctctagaactagtggatccATGAAGAGAGAGCATGGTCACCG |
|  | pGreenII 62-SK-BraRGL1-M-R | gataagcttgatatcgaattcTTATTCCACACGATTGAAACGC |
|  | Off-target1-F1 | GACTGTAAACCCGAAACCTGAC |
|  | Off-target1-R1 | TTTTAACTGTGTTTCATATAAT |
|  | Off-target1-F2 | GGGTACAAGGTTCGATCTTC |
|  | Off-target1-R2 | GAGTCACACCAGGGACCGAG |
|  | Off-target2-F1 | GATACGGAAGCTAGAGAGAG |
|  | Off-target2-R1 | GTACCAATCCCACTTGAACC |
|  | Off-target2-F2 | GGCGTGTGAGTGGGCAACTG |
|  | Off-target2-R2 | GCACAGACAAATTTTTCAGA |
|  | Off-target3-F1 | GCTGAGTTTATAGGGAAAGA |
|  | Off-target3-R1 | GGCAGTCTTGTTATCAAACA |
|  | Off-target3-F2 | GAGGAGCTGAAACCATTTTA |
|  | Off-target3-R2 | GGTCTTACATCTGGAGAGGA |
|  | Off-target4-F1 | CCTCAAGTTCGCTCACTTCA |
|  | Off-target4-R1 | CACTCGGTCTAAGCTCAAGC |
|  | Off-target4-F2 | CGGCCAACCAAGCCATCCTC |
|  | Off-target4-R2 | CTTCGATCTCACTCGGTCTA |
| BcSOC1 | BcSOC1-RT-F | ATTGTGCAGCTCAAGCAGAAG |
|  | BcSOC1-RT-R | ACTTGGGCTACTGTCCTCGT |
|  | AD-BcSOC1-F | gccatggaggccagtgaattcATGGTGAGGGGCAAAACTCA |
|  | AD-BcSOC1-R | cagctcgagctcgatggatccTCACTTTCTTGAAGAACAAGGTAACC |
|  | YNE-BcSOC1-F | cgcgccactagtggatccATGGTGAGGGGCAAAACTCA |
|  | YNE-BcSOC1-R | ggtaccctcgaggtcgacCTTTCTTGAAGAACAAGGTAACC |
|  | pGreenII 62-SK-BcSOC1-F | cgctctagaactagtggatccATGGTGAGGGGCAAAACTCA |
|  | pGreenII 62-SK-BcSOC1-R | gataagcttgatatcgaattcTCACTTTCTTGAAGAACAAGGTAACC |
| BcGASA6 | BcGASA6-RT-F | TGGAGGGGAATGCACTAGGA |
|  | BcGASA6-RT-R | TGTTTGTTGCCGTAAGTGCC |
| BcEXPA11 | BcEXPA11-RT-F | GAACTTCGCTCTCCCCAACA |
|  | BcEXPA11-RT-R | TTCCGATCTTCTCCCAAGCG |
|  | pAbAi-ProBcEXPA11-F | aaatgatgaattgaaaagcttGCATCAGGAGGATCTCCCGGTG |
|  | pAbAi-ProBcEXPA11-R | gtcgacagatccccgggtaccGATGATGATCGAGTGAAGGAGA |
|  | pGreenII-0800-ProBcEXPA11-F | ctatagggcgaattgggtaccGCATCAGGAGGATCTCCCGGTG |
|  | pGreenII-0800-ProBcEXPA11-R | tgtttttggcgtcttccatggGATGATGATCGAGTGAAGGAGA |
| BcXTH3 | BcXTH3-RT-F | CCAGTCTCTGGAACGGTGAC |
|  | BcXTH3-RT-R | CTGACAGGCCGGAAAAGTCT |
|  | pAbAi-ProBcXTH3-F | aaatgatgaattgaaaagcttATGTTTGCAGCTGGAAGAACAA |
|  | pAbAi-ProBcXTH3-R | gtcgacagatccccgggtaccCTTGGTTTGGTTTTTCTCTGAG |
|  | pGreenII-0800-ProBcXTH3-F | ctatagggcgaattgggtaccATGTTTGCAGCTGGAAGAACAA |
|  | pGreenII-0800-ProBcXTH3-R | tgtttttggcgtcttccatggCTTGGTTTGGTTTTTCTCTGAG |
| BcLFY | BcLFY-RT-F | CGAACAGTGTCGCGAGTTTC |
|  | BcLFY-RT-R | GTAGTGTCGCATCTTCGGCT |
|  | pAbAi-ProBcLFY-F | aaatgatgaattgaaaagcttATGTTTGCAGCTGGAAGAACAA |
|  | pAbAi-ProBcLFY-R | gtcgacagatccccgggtaccCTTGGTTTGGTTTTTCTCTGAG |
|  | pGreenII-0800-ProBcLFY-F | ctatagggcgaattgggtaccAACAACAAGTTAGATGTTTATT |
|  | pGreenII-0800-ProBcLFY-R | tgtttttggcgtcttccatggATTCTCTTTCTTTACTCTTTTT |
| GAPDH | GAPDH-RT-F | CAGGTTTGGAATTGTCGAGG |
|  | GAPDH-RT-R | GAGCTGTGGAAGCACCTTTC |
